# Near-highway pollutants in motor vehicle exhaust: A review of epidemiologic evidence of cardiac and pulmonary health risks

**DOI:** 10.1186/1476-069X-6-23

**Published:** 2007-08-09

**Authors:** Doug Brugge, John L Durant, Christine Rioux

**Affiliations:** 1 Tufts Community Research Center, Tufts University School of Medicine, Boston, MA, USA; 2 Department of Civil and Environmental Engineering, Tufts University, Medford, MA 02155, USA; 3 Interdisciplinary PhD Program, Tufts University, Medford, MA 02155, USA

## Abstract

There is growing evidence of a distinct set of freshly-emitted air pollutants downwind from major highways, motorways, and freeways that include elevated levels of ultrafine particulates (UFP), black carbon (BC), oxides of nitrogen (NOx), and carbon monoxide (CO). People living or otherwise spending substantial time within about 200 m of highways are exposed to these pollutants more so than persons living at a greater distance, even compared to living on busy urban streets. Evidence of the health hazards of these pollutants arises from studies that assess proximity to highways, actual exposure to the pollutants, or both. Taken as a whole, the health studies show elevated risk for development of asthma and reduced lung function in children who live near major highways. Studies of particulate matter (PM) that show associations with cardiac and pulmonary mortality also appear to indicate increasing risk as smaller geographic areas are studied, suggesting localized sources that likely include major highways. Although less work has tested the association between lung cancer and highways, the existing studies suggest an association as well. While the evidence is substantial for a link between near-highway exposures and adverse health outcomes, considerable work remains to understand the exact nature and magnitude of the risks.

## Background

Approximately 11% of US households are located within 100 meters of 4-lane highways [estimated using: [1,2]]. While it is clear that automobiles are significant sources of air pollution, the exposure of near-highway residents to pollutants in automobile exhaust has only recently begun to be characterized. There are two main reasons for this: (A) federal and state air monitoring programs are typically set up to measure pollutants at the regional, not local scale; and (B) regional monitoring stations typically do not measure all of the types of pollutants that are elevated next to highways. It is, therefore, critical to ask what is known about near-highway exposures and their possible health consequences.

Here we review studies describing measurement of near-highway air pollutants, and epidemiologic studies of cardiac and pulmonary outcomes as they relate to exposure to these pollutants and/or proximity to highways. Although some studies suggest that other health impacts are also important (e.g., birth outcomes), we feel that the case for these health effects are less well developed scientifically and do not have the same potential to drive public policy at this time. We did not seek to fully integrate the relevant cellular biology and toxicological literature, except for a few key references, because they are so vast by themselves.

We started with studies that we knew well and also searched the engineering and health literature on Medline. We were able to find some earlier epidemiologic studies based on citations in more recent articles. We include some studies that assessed motor vehicle-related pollutants at central site monitors (i.e., that did not measure highway proximity or traffic) because we feel that they add to the plausibility of the associations seen in other studies. The relative emphasis given to studies was based on our appraisal of the rigor of their methodology and the significance of their findings. We conclude with a summary and with recommendations for policy and further research.

### Motor vehicle pollution

It is well known that motor vehicle exhaust is a significant source of air pollution. The most widely reported pollutants in vehicular exhaust include carbon monoxide, nitrogen and sulfur oxides, unburned hydrocarbons (from fuel and crankcase oil), particulate matter, polycyclic aromatic hydrocarbons, and other organic compounds that derive from combustion [3-5]. While much attention has focused on the transport and transformation of these pollutants in ambient air – particularly in areas where both ambient pollutant concentrations and human exposures are elevated (e.g., congested city centers, tunnels, and urban canyons created by tall buildings), less attention has been given to measuring pollutants and exposures near heavily-trafficked highways. Several lines of evidence now suggest that steep gradients of certain pollutants exist next to heavily traveled highways and that living within these elevated pollution zones can have detrimental effects on human health.

It should be noted that many different types of highways have been studied, ranging from California "freeways" (defined as multi-lane, high-speed roadways with restricted access) to four-lane (two in each direction), variable-speed roadways with unrestricted access. There is considerable variation in the literature in defining highways and we choose to include studies in our review that used a broad range of definitions (see Table 1).

**Table 1:** Summary of near-highway pollution gradients

**Citation**	**Location**	**Highway traffic intensity^a^**	**Pollutants measured^b^**	**Observed Pollution Gradients**
Shi et al. 1999 (6)	Birmingham, UK	30,000 veh/d	UFP + FP (10-10^4 ^nm)	2–100 m ^c^
Zhu et al. 2002 (8)	Los Angeles; Freeway 710	12,180 veh/h	UFP, CO, BC	17–300 m ^c^
Zhu et al. 2002 (7)	Los Angeles; Freeway 405	13,900 veh/h	UFP, CO, BC	30–300 m ^c^
Hitchins et al. 2002 (11)	Brisbane (Austr.)	2,130–3,400 veh/h	UFP + FP (15-2 × 10^4 ^nm), PM_2.5_	15–375 m ^c^
Fischer et al. 2000 (13)	Amsterdam	<3,000–30,974 veh/d	PM_2.5_, PM_10_, PPAH, VOCs	NA
Roorda-Knape et al. 1998 (14)	Netherlands	80,000–152,000 veh/d	PM_2.5_, PM_10_, BC, VOCs, NO_2_	15–330 m ^c^
Janssen et al. 2001 (15)	Netherlands	40,000–170,000 veh/d	PM_2.5_, VOCs, NO_2_	< 400 m ^c^
Morawska et al. 1999 (12)	Brisbane (Austr.)	NA	UFP	10–210 m ^c^

^a^As defined in article cited (veh/d = vehicles per day; veh/h = vehicles per hour).

^b^UFP = ultrafine particles; FP = fine particles; PM_2.5 _= particles with aerodynamic diameter ≤ 2.5 um; PM_10 _= particles with aerodynamic diameter ≤ 10 um; BC = black carbon; PPAH = particle-bound polycyclic aromatic hydrocarbons; VOCs = volatile organic compounds

^c^Pollutant measurements were made along a transect away from the highway

NA = not applicable; measurements were not made.

It should also be noted that there may be significant heterogeneity in the types and amounts of vehicles using highways. The typical vehicle fleet in the US is composed of passenger cars, sports utility vehicles, motorcycles, pickup trucks, vans, buses, and small, medium, and large trucks. The composition and size of a fleet on a given highway may vary depending on the time of day, day of the week, and use restrictions for certain classes of vehicles. Fleets may also vary in the average age and state of repair of vehicles, the fractions of vehicles that burn diesel and gasoline, and the fraction of vehicles that have catalytic converters. These factors will influence the kinds and amounts of pollutants in tailpipe emissions. Similarly, driving conditions, fuel chemistry, and meteorology can also significantly impact emissions rates as well as the kinds and concentrations of pollutants present in the near-highway environment. These factors have rarely been taken into consideration in health outcome studies of near-highway exposure.

Based on our review of the literature, the pollutants that have most consistently been reported at elevated levels near highways include ultrafine particles (UFP), black carbon (BC), nitrogen oxides (NOx), and carbon monoxide (CO). In addition, PM_2.5_, and PM_10 _were measured in many of the epidemiologic studies we reviewed. UFP are defined as particles having an aerodynamic diameter in the range of 0.005 to 0.1 microns (um). UFP form by condensation of hot vapors in tailpipe emissions, and can grow in size by coagulation. PM_2.5 _and PM_10 _refer to particulate matter with aerodynamic diameters of 2.5 and 10 um, respectively. BC (or "soot carbon") is an impure form of elemental carbon that has a graphite-like structure. It is the major light-absorbing component of combustion aerosols. These various constituents can be measured in real time or near-real time using particle counters (UFP) and analyzers that measure light absorption (BC and CO), chemiluminescence (NOx), and weight (PM_2.5 _and PM_10_). Because UFP, NO_x_, BC, and CO derive from a common source – vehicular emissions – they are typically highly inter-correlated.

### Air pollutant gradients near highways

**Figure 1:**
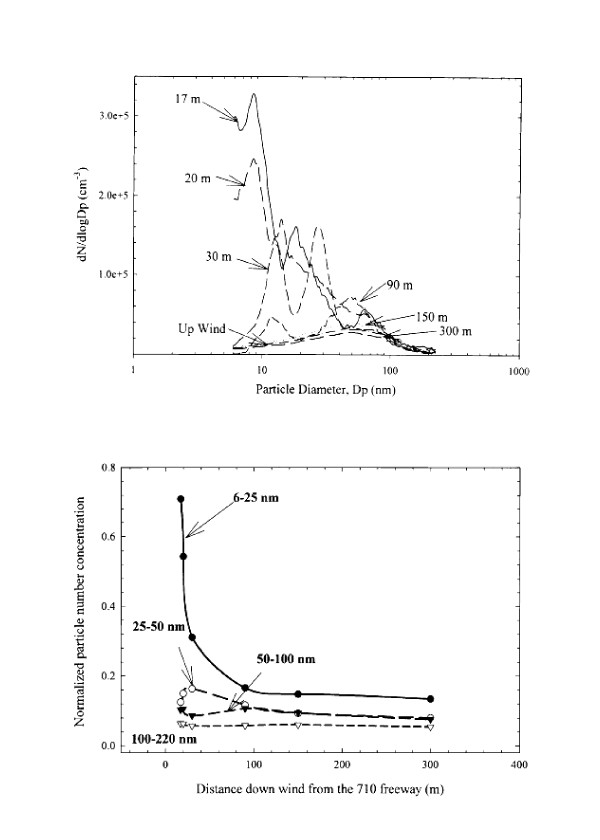
Ultrafine particle size distribution (top panel) and normalized particle number concentration for different size ranges (bottom panel) as a function of distance from a highway in Los Angeles. From Zhu et al. (8). Reprinted with permission from Elsevier.

Several recent studies have shown that sharp pollutant gradients exist near highways. Shi et al. [6] measured UFP number concentration and size distribution along a roadway-to-urban-background transect in Birmingham (UK), and found that particle number concentrations decreased nearly 5-fold within 30 m of a major roadway (>30,000 veh/d). Similar observations were made by Zhu et al. [7,8] in Los Angeles. Zhu et al. measured wind speed and direction, traffic volume, UFP number concentration and size distribution as well as BC and CO along transects downwind of a highway that is dominated by gasoline vehicles (Freeway 405; 13,900 vehicles per hour; veh/h) and a highway that carries a high percentage of diesel vehicles (Freeway 710; 12,180 veh/h). Relative concentrations of CO, BC, and total particle number concentration decreased exponentially between 17 and 150 m downwind from the highways, while at 300 m UFP number concentrations were the same as at upwind sites. An increase in the relative concentrations of larger particles and concomitant decrease in smaller particles was also observed along the transects (see Figure 1). Similar observations were made by Zhang et al. [9] who demonstrated "road-to-ambient" evolution of particle number distributions near highways 405 and 710 in both winter and summer. Zhang et al. observed that between 30–90 m downwind of the highways, particles grew larger than 0.01 um due to condensation, while at distances >90 m, there was both continued particle growth (to >0.1 um) as well as particle shrinkage to <0.01 um due to evaporation. Because condensation, evaporation, and dilution alter size distribution and particle composition, freshly-emitted UFP near highways may differ in chemical composition from UFP that has undergone atmospheric transformation during transport to downwind locations [10].

Two studies in Brisbane (Australia) highlight the importance of wind speed and direction as well as contributions of pollutants from nearby roadways in tracking highway-generated pollutant gradients. Hitchins et al. [11] measured the mass concentrations of 0.1–10 um particles as well as total particle number concentration and size distribution for 0.015–0.7 um particles near highways (2,130–3,400 veh/h). Hitchens et al. observed that the distance from highways at which number and mass concentrations decreased by 50% varied from 100 to 375 m depending on the wind speed and direction. Morawska et al. [12] measured the changes in UFP number concentrations along horizontal and vertical transects near highways to distinguish highway and normal street traffic contributions. It was observed that UFP number concentrations were highest <15 m from highways, while 15–200 m from highways there was no significant difference in UFP number concentrations along either horizontal or vertical transects – presumably due to mixing of highway pollutants with emissions from traffic on nearby, local roadways.

In addition to UFP, other pollutants – such as PM_2.5_, PM_10_, NO_2 _(nitrogen dioxide), VOCs (volatile organic compounds), and particle-bound polycyclic aromatic hydrocarbons (PPAH) – have been studied in relation to heavily-trafficked roadways. Fischer et al. [13] measured PM_2.5_, PM_10_, PPAH, and VOC concentrations outside and inside homes on streets with high and low traffic volumes in Amsterdam (<3,000–30,974 veh/d). In this study, PPAH and VOCs were measured using methods based on gas chromatography. Fischer et al. found that while PM_2.5 _and PM_10 _mass concentrations were not specific indicators of traffic-related air pollution, PPAH and VOC levels were ~2-fold higher both indoor and outdoor in high traffic areas compared to low traffic areas. Roorda-Knape et al [14] measured PM_2.5_, PM_10_, black smoke (which is similar to BC), NO_2_, and benzene in residential areas <300 m from highways (80,000–152,000 veh/d) in the Netherlands. Black smoke was measured by a reflectance-based method using filtered particles; benzene was measured using a method based on gas chromatography. Roorda-Knape et al reported that outdoor concentrations of black smoke and NO_2 _decreased with distance from highways, while PM_2.5_, PM_10_, and benzene concentrations did not change with distance. In addition, Roorda-Knape et al. found that indoor black smoke concentrations were correlated with truck traffic, and NO_2 _was correlated with both traffic volume and distance from highways. Janssen et al. [15] studied PM_2.5_, PM_10_, benzene, and black smoke in 24 schools in the Netherlands and found that PM_2.5 _and black smoke increased with truck traffic and decreased with distance from highways (40,000–170,000 veh/d).

In summary, the literature shows that UFP, BC, CO and NOx are elevated near highways (>30,000 veh/d), and that other pollutants including VOCs and PPAHs may also be elevated. Thus, people living within about 30 m of highways are likely to receive much higher exposure to traffic-related air pollutants compared to residents living >200 m (+/- 50 m) from highways.

### Cardiovascular health and traffic-related pollution

Results from clinical, epidemiological, and animal studies are converging to indicate that short-term and long-term exposures to traffic-related pollution, especially particulates, have adverse cardiovascular effects [16-18]. Most of these studies have focused on, and/or demonstrated the strongest associations between cardiovascular health outcomes and particulates by weight or number concentrations [19-21] though CO, SO_2_, NO_2_, and BC have also been examined. BC has been shown to be associated with decreases in heart rate variability (HRV) [22,23] and black smoke and NO_2 _shown to be associated with cardiopulmonary mortality [24].

Short-term exposure to fine particulate pollution exacerbates existing pulmonary and cardiovascular disease and long-term repeated exposures increases the risk of cardiovascular disease and death [25,26].

Though not focused on near-highway pollution, two large prospective cohort studies, the Six-Cities Study [27] and the American Cancer Society (ACS) Study [28] provided the groundwork for later research on fine particulates and cardiovascular disease. Both of these studies found associations between increased levels of exposure to ambient PM and sulfate air pollution recorded at central city monitors and annual average mortality from cardiopulmonary disease, which at the time combined cardiovascular and pulmonary disease other than lung cancer. The Six-Cities Study examined PM_2.5 _and PM_10/15_. The ACS study examined PM _2.5_. Relative risk ratios of mortality from cardiopulmonary disease comparing locations with the highest and lowest fine particle concentrations (which had differences of 24.5 and 18.6 ug/m^3 ^respectively) were 1.37 (1.11, 1.68) and 1.31 (1.17, 1.46) in the Six Cities and ACS studies, respectively. These analyses controlled for many confounders, including smoking and gas stoves but not other housing conditions or time spent at home. The studies were subject to intensive replication, validation, and reanalysis that confirmed the original findings. PM_2.5 _generally declined following implementation of new US Environmental Protection Agency standards in 1997 [17,29], yet since that time studies have shown elevated health risks due to long-term exposures to the 1997 PM threshold concentrations [29,30].

Much of the epidemiological research has focused on assessing the early physiological responses to short-term fluctuations in air pollution in order to understand how these exposures may alter cardiovascular risk profiles and exacerbate cardiovascular disease [31]. Heart rate variability, a risk factor for future cardiovascular outcomes, is altered by traffic-related pollutants particularly in older people and people with heart disease [22,23,32]. With decreased heart rate variability as the adverse outcome, negative associations between HRV and particulates were strongest for the smallest size fraction studied [33] (PM0.3–1.0); [34] (PM0.02–1). In two studies that included other pollutants, black carbon, an indicator of traffic particles, also elicited a strong association with both time and frequency domain HRV variables; associations were also strong for PM2.5 for both time and frequency HRV variables in the Adar et al study [[23]; this and subsequent near highway studies are summarized in Table 2], however, PM2.5 was not associated with frequency domain variables in the Schwartz et al. study [22].

**Table 2:** Summary of near-highway health effects studies

**Citation**	**Location**	**Highway traffic intensity^a^**	**Pollutants measured^b^**	**Distance from highway**	**Health Outcomes**	**Statistical association^e^**
Schwartz et al. 2005 (22)	Boston	NA	PM_2.5_, BC, CO	NA	Heart rate variability	Decreases in measures of heart rate variability
Adar et al. 2007 (23)	St. Louis, Missouri	NA	PM_2.5_, BC, UFP	On highway in busses	Heart rate variability	Decreases in measures of heart rate variability
Hoek et al. 2002 (24)	Netherlands	NA	BC, NO_2_	Continuous ^d^	Cardio-pulmonary mortality, lung cancer	1.41 OR for living near road
Tonne et al. 2007 (41)	Worchester, Mass.	NA	PM_2.5_	Continuous ^d^	Acute myocardial infarction (AMI)	5% increase in odds of AMI
Venn et al. 2001 (49)	Nottingham, UK	NA	NA	Continuous ^d^	Wheezing in children	1.08 OR for living w/in 150 m of road
Nicolai et al. 2003 (58)	Munich, Germany	>30,000 veh/d	Soot, benzene, NO_2_	Traffic counts within 50 m of house	Asthma, respiratory symptoms, allergy	1.79 OR for asthma and high traffic volume
Gauderman et al. 2005 (65)	Southern California		NO_2_	Continuous ^d^	Asthma, respiratory symptoms	Increased asthma closer to freeways
McConnell et al. 2006 (57)	Southern California	NA	NA	Continuous ^d^	Asthma	Large risk for children living w/in 75 m of road
Ryan, et al. 2007 (59)	Cincinnati, Ohio	> 1,000 trucks/d	PM2.5	400 m	Wheezing in children	NA
Kim et al. 2004 (60)	San Francisco	90,000 – 210,000 veh/d	PM, BC, NO_x_	School sites	Childhood asthma	1.07 OR for high levels of NO_x_
Wjst et al. 1993 (68)	Munich, Germany	7,000–125,000 veh/d	NO_x_, CO	School sites	Asthma, bronchitis	Several statistical associations found
Brunekreef et al. 1997 (69)	Netherlands	80,000 – 152,000 veh/d	PM_10_, NO_2_	Continuous^d^	Lung function	Decreased FEV with proximity to high truck traffic
Janssen et al. 2003 (74)	Netherlands	30,000–155,000 veh/d	PM_2.5_, NO_2_, benzene	< 400 m ^c^	Lung function, respiratory symptoms	No association with lung function
Peters et al. 1999 (82)	Southern California	NA	PM_10_, NO_2_	NA	Asthma, bronchitis, cough, wheeze	1.54 OR of wheeze for boys with exposure to NO_2_
Brauer et al. 2007 (67)	Netherlands	Highways and streets	PM_2.5_, NO_2_, soot	Modeled exposure	Asthma, allergy, bronchitis, respiratory symptoms	Strongest association was with food allergies
Visser et al. 2004 (91)	Amsterdam	> 10,000 veh/d	NA	NA	Cancer	Multiple associations
Vineis et al. 2006 (87)	10 Eurpoean countries	NA	PM_10_, NO_2_, SO_2_	NA	Cancer	1.46 OR near heavy traffic, 1.30 OR for high exposure to NO_2_
Gauderman et al. 2007 (73)	Southern California	NA	PM_10_, NO_2_	Continuous^d^	Lung Function	Decreased FEV for those living near freeway

^a^As defined in article cited (veh/d = vehicles per day; veh/h = vehicles per hour).

^b^UFP = ultrafine particles; FP = fine particles; PM_2.5 _= particles with aerodynamic diameter ≤ 2.5 um; PM_10 _= particles with aerodynamic diameter ≤ 10 um; BC = black carbon; PPAH = particle-bound polycyclic aromatic hydrocarbons; VOCs = volatile organic compounds

^c^Pollutant measurements were made along a transect away from the highway

^d^Proximity of each participant to a major road was calculated using GIS software

^e^Statistical association between proximity to highway or exposure to traffic-generated pollutants and measured health outcomes

NA = not applicable; measurements were not made.

Several studies show that exposure to PM varies spatially within a city [35-37], and finer spatial analyses show higher risks to individuals living in close proximity to heavily trafficked roads [18,37]. A 2007 paper from the Woman's' Health Initiative used data from 573 PM_2.5 _monitors to follow over 65,000 women prospectively. They reported very high hazard ratios for cardiovascular events (1.76; 95% CI, 1.25 to 2.47) possibly due to the fine grain of exposure monitoring [18]. In contrast, studies that relied on central monitors [27,28] or interpolations from central monitors to highways are prone to exposure misclassification because individuals living close to highways will have a higher exposure than the general area. A possible concern with this interpretation is that social gradients may also situate poorer neighborhoods with potentially more susceptible populations closer to highways [38-40].

At a finer grain, Hoek et al. [24] estimated home exposure to nitrogen dioxide (NO_2_) and black smoke for about 5,000 participants in the Netherlands Cohort Study on Diet and Cancer. Modeled exposure took into consideration proximity to freeways and main roads (100 m and 50 m, respectively). Cardiopulmonary mortality was associated with both modeled levels of pollutants and living near a major road with associations less strong for background levels of both pollutants. A case-control study [41], found a 5% increase in acute myocardial infarction associated with living within 100 m of major roadways. A recent analysis of cohort data found that traffic density was a predictor of mortality more so than was ambient air pollution [42]. There is a need for studies that assess exposure at these scales, e.g., immediate vicinity of highways, to test whether cardiac risk increases still more at even smaller scales.

Although we cannot review it in full here, we note that evidence beyond the epidemiological literature support the contention that PM_2.5 _and UFP (a sub-fraction of PM_2.5_) have adverse cardiovascular effects [16,17]. PM_2.5 _appears to be a risk factor for cardiovascular disease via mechanisms that likely include pulmonary and systemic inflammation, accelerated atherosclerosis and altered cardiac autonomic function [17,22,43-46]. Uptake of particles or particle constituents in the blood can affect the autonomic control of the heart and circulatory system. Black smoke, a large proportion of which is derived from mobile source emissions [30], has a high pulmonary deposition efficiency, and due to their surface area-to-volume ratios can carry relatively more adsorbed and condensed toxic air pollutants (e.g., PPAH) compared to larger particles [17,47,48]. Based on high particle numbers, high lung deposition efficiency and surface chemistry, UFP may provide a greater potential than PM_2.5 _for inducing inflammation [10]. UFPs have high cytotoxic reactive oxygen species (ROS) activity, through which numerous inflammatory responses are induced, compared to other particles [10]. Chronically elevated UFP levels such as those to which residents living near heavily trafficked roadways are likely exposed can lead to long-term or repeated increases in systemic inflammation that promote arteriosclerosis [18,29,34,37].

### Asthma and highway exposures

Evidence that near highway exposures present elevated risk is relatively well developed with respect to child asthma studies. These studies have evolved over time with the use of different methodologies. Studies that used larger geographic frames and/or overall traffic in the vicinity of the home or school [49-52] or that used self-report of traffic intensity [53] found no association with asthma prevalence. Most recent child asthma studies have, instead, used increasingly narrow definitions of proximity to traffic, including air monitoring or modeling) and have focused on major highways instead of street traffic [54-59]. All of these studies have found statistically significant associations between the prevalence of asthma or wheezing and living very close to high volume vehicle roadways. Confounders considered included housing conditions (pests, pets, gas stoves, water damage), exposure to tobacco smoke, various measures of socioeconomic status (SES), age, sex, and atopy, albeit self-reported and not all in a single study.

Multiple studies have found girls to be at greater risk than boys for asthma resulting from highway exposure [55,57,60]. A recent study also reports elevated risk only for children who moved next to the highway before they were 2 years of age, suggesting that early childhood exposure may be key [57]. The combined evidence suggests that living within 100 meters of major highways is a risk factor, although smaller distances may also result in graded increases in risk. The neglect of wind direction and the absence of air monitoring from some studies are notable missing factors. Additionally, recent concerns have been raised that geocoding (attaching a physical location to addresses) could introduce bias due to inaccuracy in locations [61].

Studies that rely on general area monitoring of ambient pollution and assess regional pollution on a scale orders of magnitude greater than the near-roadway gradients have also found associations between traffic generated pollution (CO and NOx) and prevalence of asthma [62] or hospital admission for asthma [63]. Lweguga-Mukasa et al. [64] monitored air up and down wind of a major motor vehicle bridge complex in Buffalo, NY and found that UFP were higher downwind, dropping off with distance. Their statistical models did not, however, support an association of UFP with asthma. A study in the San Francisco Bay Area measured PM_2.5_, BC and NO_X _over several months next to schools and found both higher pollution levels downwind from highways and a linear association of BC with asthma in long-term residents [60].

Gauderman et al. [65] measured NO_2 _next to homes of 208 children. They found an odds ratio (OR) of 1.83 (confidence interval (CI): 1.04–3.22) for outdoor NO_2 _(probably a surrogate for total highway pollution) and lifetime diagnosis of asthma. They also found a similar association with distance from residence to freeway. Self-report was used to control for numerous confounders, including tobacco smoke, SES, gas stoves, mildew, water damage, cockroaches and pets which did not substantially affect the association. Gauderman's study suggests that ambient air monitoring at the residence substantially increases statistical power to detect association of asthma with highway exposures.

Modeling of elemental carbon attributable to traffic near roadways based on ambient air monitoring of PM_2.5 _has recently emerged as a viable approach and a study using this method found an association with infant wheezing. The modeled values appear to be better predictors than proximity. Elevation of the residence relative to traffic was also an important factor in this study [66]. A 2007 paper reported on modeled NO_2_, PM_2.5 _and soot and the association of these values with asthma and various respiratory symptoms in the Netherlands [67]. While finding modest statistically significant associations for asthma and symptoms, it is somewhat surprising that they found stronger associations for development of sensitization to food allergens.

### Pediatric lung function and traffic-related air pollution

Studies of association of children's lung function with traffic pollutants have used a variety of measures of exposure, including: traffic density, distance to roadways, area (city) monitors, monitoring at the home or school and personal monitoring. Studies have assessed both chronic effects on lung development and acute effects and have been both cross-sectional and longitudinal. The wide range of approaches somewhat complicates evaluation of the literature.

Traffic density in school districts in Munich was associated with decreases in forced vital capacity (FVC), forced expiratory volume in 1 second (FEV_1_), FEV1/FVC and other measures, although the 2-kilometer (km) areas, the use of sitting position for spirometry and problems with translation for non-German children were limitations [68]. Brunekreef et al. [69] used distance from major roadways, considered wind direction and measured black smoke and NO2 inside schools. They found the largest decrements in lung function in girls living within 300 m of the roadways.

A longitudinal study of children (average age at start = 10 years) in Southern California reported results at 4 [70] and 8 years [71]. Multiple air pollutants were measured at sites in 12 communities. Due to substantial attrition, only 42% of children enrolled at the start were available for the 8-year follow-up. Substantially lower growth in FEV_1 _was associated with PM_10_, NO_2_, PM_2.5_, acid vapor and elemental carbon at 4 and at 8 years. The analysis could not indicate whether the effects seen were reversible or not [72]. In 2007, it was reported from this same cohort that living within 500 m of a freeway was reported to be associated with reduced lung function [73].

A Dutch study [74] measured PM_2.5_, NO_2_, benzene and EC for one year at 24 schools located within 400 m of major roadways. While associations were seen between symptoms and truck traffic and measured pollutants, there was no significant association between any of the environmental measures and FVC < 85% or FEV_1 _< 85%. Restricting the analysis to children living within 500 m of highways generally increased ORs.

Personal exposure monitoring of NO_2 _as a surrogate for total traffic pollutants with 298 Korean college students found statistically significant associations with FEV_1_, FEV_1_/FVC, and forced expiratory volume between 25 and 75% (FEV_25–75_), but not with FVC. The multivariate regression model presented suggests that FEV_25–75 _was the outcome measure that most clearly showed an effect [75]. Cross-sectional studies of children in Korea [76] and France [77] also indicate that lung function is diminished in association with area pollutants that largely derive from traffic.

Time series studies suggest there are also acute effects. A study of 19 asthmatic children measured PM via personally carried monitors, at homes and at central site monitors. The study found deficits in FEV_1 _that were associated with PM, although many sources besides traffic contributed to exposure. In addition, the results suggest that ability to see associations with health outcomes improves at finer scale of monitoring [78]. PM was associated with reduced FEV_1 _and FVC in only the asthmatic subset of children in a Seattle study [79]. Studies have also seen associations between PM and self reported peak flow measurements [80,81] and asthmatic symptoms [82].

### Cancer and near highway exposures

As noted above, both the Six-Cities Study [27] and the American Cancer Society (ACS) Study [28] found associations between PM and lung cancer. Follow-up studies using the ACS cohort [29,37] and the Six-Studies cohort [83] that controlled for smoking and other risk factors also demonstrated significant associations between PM and lung cancer. The original studies were subject to intensive replication, validation, and re-analysis which confirmed the original findings [84].

The ASHMOG study [85] was designed to look specifically at lung cancer and air pollution among Seventh-day Adventists in California, taking advantage of their low smoking rates. Air pollution was interpolated to centroids of zip codes from ambient air monitoring stations. Highway proximity was not considered. The study found associations with ozone (its primary pollutant of consideration), PM10 and SO2. Notably, these are not the pollutants that would be expected to be substantially elevated immediately adjacent to highways.

A case control study of residents of Stockholm, Sweden modeled traffic-related NO2 levels at their homes over 30 years and found that the strongest association involved a 20 year latency period [86]. Another case control study drawn from the European Prospective Investigation on Cancer and Nutrition found statistically significantly elevated ORs for lung cancer with proximity to heavy traffic (>10,000 cars per day) as well as for NO_2 _and PM_10 _at nearby ambient monitoring stations [87]. Nafstad et al. [88] used modeled NO_2 _and SO_2 _concentrations at the homes of over 16,000 men in Oslo to test associations with lung cancer incidence. The models included traffic and point sources. The study found small, but statistically significant associations between NO_2 _and lung cancer. Problems that run through all these studies are weak measures of exposure to secondhand tobacco smoke, the use of main roads rather than highways as the exposure group and modeled rather than measured air pollutants.

A study of regional pollution in Japan and a case control study of more localized pollution in a town in Italy also found associations between NO_2 _and lung cancer and PM and lung cancer [89,90]. On the other hand, a study that calculated SIRs for specific cancers across lower and higher traffic intensity found little evidence of an association with a range of cancers [91].

The plausibility of near-highway pollution causing lung cancer is bolstered by the presence of known carcinogens in diesel PM. The US EPA has concluded after reviewing the literature that diesel exhaust is "likely to be carcinogenic to humans by inhalation" [92]. An interesting study of UFP and DNA damage adds credibility to an association with cancer [93]. This study had participants bicycle in traffic in Copenhagen and measured personal exposure to UFP and DNA oxidation and strand breaks in mononuclear blood cells. Bicycling in traffic increased UFP exposure and oxidative damage to DNA, thus demonstrating an association between DNA damage and UFP exposure *in vivo*.

### Policy and research recommendations

Based on the literature reviewed above it is plausible that gradients of pollutants next to highways carry elevated health risks that may be larger than the risks of general area ambient pollutants. While the evidence is considerable, it is not overwhelming and is weak in some areas. The strongest evidence comes from studies of development of asthma and reduction of lung function during childhood, while the studies of cardiac health risk require extrapolation from area studies of smaller and larger geographic scales and inference from toxicology laboratory investigations. The lung cancer studies, because they include pollutants such as O_3 _that are not locally concentrated, are not particularly strong in terms of the case for near-highway risk. There is a need for lung cancer research that uses major highways rather than heavily trafficked roads as the environmental exposure.

While more studies of asthma and lung function in children are needed to confirm existing findings, especially studies that integrate exposure at school, home and during commuting, to refine our knowledge about the association, we would point to the greater need for studies of cardiac health and lung cancer and their association with near highway exposures as the primary research areas needing to be developed. Many of the studies of PM and cardiac or pulmonary health have focused on mortality. Near highway mortality studies may be possible, but would be lengthy if they were initiated as prospective cohorts. Other possibilities include retrospective case control studies of mortality, cross sectional studies or prospective studies that have end points short of mortality, such as biological markers of disease. For all health end points there is a need for studies that adequately address the possible confounding of SES with proximity to highways. There is good reason to think that property values decline near highways and that control for SES by, for example, income, may be inadequate.

Because of the incomplete development of the science regarding the health risks of near highway exposures and the high cost and implication of at least some possible changes in planning and development, policy decisions are complicated. The State of California has largely prohibited siting of schools within 500 feet of freeways (SB 352; approved by the governor October 2, 2003). Perhaps this is a viable model for other states or for national-level response. As it is the only such law of which we are aware, there may be other approaches that will be and should be tried. One limitation of the California approach is that it does nothing to address the population already exposed at schools currently cited near freeways and does not address residence near freeways.

## Conclusion

The most susceptible (and overlooked) population in the US subject to serious health effects from air pollution may be those who live very near major regional transportation route, especially highways. Policies that have been technology based and regional in orientation do not efficiently address the very large exposure and health gradients suffered by these populations. This is problematic because even regions that EPA has deemed to be in regional PM "attainment" still include very large numbers of near highway residents who currently are not protected. There is a need for more research, but also a need to begin to explore policy options that would protect the exposed population.

## Abbreviations

UFP = ultra fine particles

BC = black carbon

NO_2 _= nitrogen dioxide

NOx = oxides of nitrogen

CO = carbon monoxide

PM = particulate matter

PM_2.5 _= particulate matter less than 2.5 um

PM_10 _= particulate matter less than 10 um

PPAH = particle bound polyaromatic hydrocarbons

EC = elemental carbon

VOC = volatile organic compounds

SO_2 _= sulfur dioxide

ACS = American Cancer Society

SES = socioeconomic status

EPA = Environmental Protection Agency

OR = odds ratio

FEV_1 _= forced expiratory volume in 1 second

FEV_1_/FVC = ratio of FEV_1 _and forced vital capacity

FEV_25–75 _= forced expiratory volume between 25 and 75

FVC = forced vital capacity

ug/m^3 ^= micrograms per cubic meter of air

m = meters

um = micrometers

veh/d = vehicles per day

veh/h = vehicles per hour

## Competing interests

The author(s) declare that they have no competing interests.

## Authors' contributions

DB took the lead on the manuscript. He co-wrote the background and wrote the sections on asthma, lung function and cancer and the conclusions. JLD wrote the section on air pollutants near roadways and contributed substantially to the background. CR wrote the section on cardiovascular health. All authors participated in editing and refining the manuscript and all read it multiple times, including the final version.
